# Copy Number Variation Analysis in Familial *BRCA1/2*-Negative Finnish Breast and Ovarian Cancer

**DOI:** 10.1371/journal.pone.0071802

**Published:** 2013-08-13

**Authors:** Kirsi M. Kuusisto, Oyediran Akinrinade, Mauno Vihinen, Minna Kankuri-Tammilehto, Satu-Leena Laasanen, Johanna Schleutker

**Affiliations:** 1 Institute of Biomedical Technology/BioMediTech, University of Tampere and Fimlab Laboratories, Tampere, Finland; 2 Department of Experimental Medical Science, Lund University, Lund, Sweden; 3 Department of Clinical Genetics, Turku University Hospital, Turku, Finland; 4 Department of Pediatrics, Genetics Outpatient Clinic, and Department of Dermatology, Tampere University Hospital, Tampere, Finland; 5 Department of Medical Biochemistry and Genetics, Institute of Biomedicine, University of Turku, Turku, Finland; Health Canada and University of Ottawa, Canada

## Abstract

**Background:**

Inherited factors predisposing individuals to breast and ovarian cancer are largely unidentified in a majority of families with hereditary breast and ovarian cancer (HBOC). We aimed to identify germline copy number variations (CNVs) contributing to HBOC susceptibility in the Finnish population.

**Methods:**

A cohort of 84 HBOC individuals (negative for *BRCA1/2-*founder mutations and pre-screened for the most common breast cancer genes) and 36 healthy controls were analysed with a genome-wide SNP array. CNV-affecting genes were further studied by Gene Ontology term enrichment, pathway analyses, and database searches to reveal genes with potential for breast and ovarian cancer predisposition. CNVs that were considered to be important were validated and genotyped in 20 additional HBOC individuals (6 CNVs) and in additional healthy controls (5 CNVs) by qPCR.

**Results:**

An intronic deletion in the *EPHA3* receptor tyrosine kinase was enriched in HBOC individuals (12 of 101, 11.9%) compared with controls (27 of 432, 6.3%) (OR = 1.96; *P* = 0.055). *EPHA3* was identified in several enriched molecular functions including receptor activity. Both a novel intronic deletion in the *CSMD1* tumor suppressor gene and a homozygous intergenic deletion at 5q15 were identified in 1 of 101 (1.0%) HBOC individuals but were very rare (1 of 436, 0.2% and 1 of 899, 0.1%, respectively) in healthy controls suggesting that these variants confer disease susceptibility.

**Conclusion:**

This study reveals new information regarding the germline CNVs that likely contribute to HBOC susceptibility in Finland. This information may be used to facilitate the genetic counselling of HBOC individuals but the preliminary results warrant additional studies of a larger study group.

## Introduction

Breast cancer (BC) is the most common cancer among women in western countries, including Finland. Inherited BC risk is known to be associated with rare, highly penetrant variants, mainly single nucleotide polymorphisms (SNPs) and small insertions and deletions (indels) in *BRCA1* and *BRCA2,* which account for nearly 20% of hereditary breast and/or ovarian cancer (HBOC) cases in Finland [1]–[3]. Additionally, variants in other *BRCA1/2* interacting genes, including *CHEK2, PALB2, RAD51C*, and *Abraxas*, are known to account for a low proportion of HBOC susceptibility in the Finnish population [4]–[7].

In addition to SNPs and small indels, copy number variations (CNVs) contribute to susceptibility to complex diseases and disorders [8]. A CNV is a segment of DNA (1 kb or larger) that presents an altered copy number compared with the reference genome [9]. Depending on the location, CNVs may affect target gene expression through a dosage effect or by disrupting gene regulatory elements [10]. CNVs were initially associated with neurological disorders, but studies have demonstrated the role of CNVs also in other diseases, including several cancers [11]–[15].

Despite the fact that several heritable risk factors for breast and ovarian cancer have been recognised, in the majority (up to 80%) of HBOC families, inherited risk is likely explained by yet unknown factors, which makes genetic counselling and clinical surveillance challenging. The contribution of rare germline CNVs to breast and ovarian cancer susceptibility has also been established in the Finnish population, but their role is mostly unexplored [16], [17]. Therefore, new information regarding germline CNVs and their role in HBOC predisposition is needed to identify CNVs that may be used clinically to facilitate the genetic counselling of HBOC families.

To determine additional genetic factors contributing to HBOC susceptibility in the Finnish population and gain new information for genetic counselling, we analysed germline CNVs in a cohort of 84 well-characterised HBOC *BRCA1/2*-founder mutation-negative Finnish individuals who have been pre-screened for the most common high- and moderate-penetrant genes [18].

## Materials and Methods

### Study Material

Index individuals from 84 HBOC families were collected from the Tampere University Hospital Genetics Outpatient Clinic between January 1997 and May 2008. Individuals were selected according to previously reported high-risk hereditary BC criteria [18]. All individuals had been determined to be founder mutation-negative by minisequencing the 28 previously known Finnish *BRCA1/2* mutations and a protein truncation test (PTT) for *BRCA1* exon 11 and *BRCA2* exons 10 and 11. Eighty-one of the individuals included in this study have previously been characterised and screened for germline alterations in seven known BC-associated genes [18]. In addition, the index individuals from three additional HBOC families were included (described in File S1). For CNV validation analysis, index individuals from 20 additional HBOC families, collected from Turku University Hospital Genetics Outpatient Clinic between 2007 and 2011 were utilised. Clinical characteristics of the 20 additional HBOC individuals (negative for *BRCA1/2*-mutations) are described in File S2. As controls, 905 DNA samples from anonymous healthy females, collected from the Finnish Red Cross, were used. All of the HBOC individuals studied have been informed of the analyses, and they have given written consent to use their existing DNA samples. Permission for the research project has been received from the Ethical Committees of Tampere and Turku University Hospitals and the National Authority for Medicolegal Affairs.

### Copy Number Variation Analysis

The DNA samples from 84 HBOC individuals and 36 controls were genotyped by using the genome-wide SNP array HumanCytoSNP-12 v.2.1 Beadchip (Illumina, Inc, San Diego, CA, USA), which targets regions of known cytogenetic importance. Sample preparation was performed according to the Infinium II assay protocol (Illumina, Inc, San Diego, CA, USA) at the Institute for Molecular Medicine, Finland. Log R Ratios (LRRs), B Allele frequencies (BAF), and X and Y channel intensities for each sample were exported from normalised Illumina data using GenomeStudio software (GSGTv1.7.4) to perform CNV calling. All of the samples had call rates greater than 99.5%. High sample quality was ensured by applying previously reported quality criteria [19]. Thus, 81 HBOC individuals and 35 controls were suitable for analysis. CNV calling was performed with the PennCNV (2009Aug27) program [19]. Additionally, two other programs, QuantiSNP v2.3 [20] and cnvPartition v3.1.6 (Illumina Inc, San Diego, CA, USA) were used to confirm the PennCNV results when selecting CNVs for validation. Programs were used with default parameters. CNVs spanning less than three SNPs were filtered out.

### Statistical Analyses

CNV distribution and median lengths were compared between HBOC individuals and controls using the Wilcoxon test (R v2.15.2, R Development Core Team, R Foundation for Statistical Computing, Vienna, Austria). CNV carrier frequencies between HBOC individuals and controls were compared with the Fisher’s exact or *χ* ^2^ tests (R v2.15.2 and PLINK v.1.07 [21]). All *P*-values were two-sided. A *P*-value<0.05 was considered statistically significant. Furthermore, a VCD package was implemented in R to estimate the numerical values of the odds ratios for enrichment analysis in case a non-numerical value was returned from the Fisher’s exact test [22].

### CNV Validation and Genotyping by Quantitative Real-time PCR (qPCR)

Selected CNVs were validated (6 CNVs) and genotyped in 20 additional HBOC individuals (6 CNVs) and in 299–869 additional healthy female controls (5 CNVs) by TaqMan® Copy Number Assays and TaqMan® real-time PCR, respectively, on an ABI PRISM 7900 sequence detection system (Applied Biosystems, Foster City, CA, USA). The following pre-designed TaqMan® Copy Number Assays were used: Hs04703682_cn (2q34), Hs03458738_cn (3p11.1), Hs03253932_cn (5q15), Hs06178677_cn (8p23.2), Hs02640223_cn (17q21.31), and Hs04482315_cn (19q13.41). As an internal standard, a TaqMan® RNaseP Reference Assay (Applied Biosystems, Part Number 4403326) was run with the pre-designed TaqMan® Copy Number Assays in a duplex, real-time PCR reaction (see File S3 for more details).

### Data Analysis

Identified CNVs were queried for overlap with the Database of Genomic Variants (DGV), Toronto (http://projects.tcag.ca/variation/) using NCBI Genome Build 36 (hg 18). A CNV locus was considered novel if it did not overlap with any of the established CNV loci in the DGV. CNVs were annotated using NCBI RefSeq genes (http://www.ncbi.nlm.nih.gov/RefSeq/) to identify genes/exons overlapping the observed CNV loci. For intergenic CNVs, the loci were expanded upstream and downstream of the CNV to identify neighbouring genes.

Enrichment analyses, including Gene Ontology (GO) terms, KEGG pathways, Pathway Commons, and Wikipathways, were performed for CNV-affecting genes to reveal common functions of the gene products using the Web-based Gene Set Analysis Toolkit V2 (WebGestalt2) [23]. Furthermore, CNV-affected genes were queried for overlap against genes listed in the NCBI Online Mendelian Inheritance in Man (OMIM) database (http://www.ncbi.nlm.nih.gov/omim) to identify genomic loci associated with genetic disorders. In addition, a Genetic Association Database (GAD) (http://geneticassociationdb.nih.gov/) search was performed to identify genes analysed in previous association studies for complex diseases and disorders.

## Results

We performed genome-wide CNV analysis with a SNP array targeting regions of known cytogenetic importance for individuals from 84 Finnish HBOC families and 36 healthy controls. After applying the quality control criteria, 81 HBOC individuals and 35 controls (n = 116) were included in the data analysis. The aim of this study was to identify germline CNVs contributing to HBOC susceptibility in Finnish families.

The PennCNV program was used to detect 545 autosomal CNVs at 273 different genomic regions in HBOC individuals and controls (n = 116). All of the identified CNVs are presented in detail in Table S1. A summary of the CNVs identified by PennCNV are shown in Table 1. The most important observations are that the average number of CNVs was slightly higher in HBOC individuals compared with controls, and deletions were more frequent in HBOC individuals. There was no statistically significant difference in the median size the CNVs between the HBOC individuals and controls (52.3 kb vs. 50.5 kb; *P* = 0.90). However, the median deletion size in HBOC individuals was smaller compared with the controls (39.2 kb vs. 56.8 kb; *P* = 0.07). In contrast, the median duplication size was significantly larger (*P* = 0.01) in HBOC individuals compared with controls (68.7 kb vs. 47.5 kb).

**Table 1:** Summary of the identified copy number variations (CNVs) by PennCNV in 81 hereditary breast and/or ovarian cancer (HBOC) individuals and 35 controls.

	Average noper sample	Median size (kb)	Gene-affecting (%)	Novel CNVs (%)
**All CNVs (n = 545)**				
HBOC individuals	392/81 (4.8)	52.3	228/392 (0.58)	37/392 (0.09)
Controls	153/35 (4.4)	50.5	85/153 (0.56)	11/153 (0.07)
HBOC individuals only	215/81 (2.7)	52.5	141/215 (0.66)	36/215 (0.17)
**Deletions (n = 300)**				
HBOC individuals	222/81 (2.7)	39.2	109/222 (0.56)	30/222 (0.14)
Controls	78/35 (2.2)	56.8	34/78 (0.44)	4/78 (0.05)
HBOC individuals only	116/81 (1.4)	34.6	72/116 (0.62)	29/116 (0.25)
**Duplications (n = 245)**				
HBOC individuals	170/81 (2.1)	68.7	119/170 (0.70)	7/170 (0.04)
Controls	75/245 (2.1)	47.5	51/75 (0.68)	7/75 (0.09)
HBOC individuals only	99/245 (1.2)	60.8	69/99 (0.70)	7/99 (0.07)

Abbreviations: no = number.

Annotation of all of the 545 CNVs against the genes in the NCBI RefSeq database revealed 313 (57.4%) gene-affecting CNVs (Table 1). Most importantly, gene-affecting deletions were more common in HBOC individuals compared with controls (Table 1). The identified CNVs were compared with healthy control sample data collected in the Database of Genomic Variants. The main observation was that the proportion of novel deletions to all deletions in HBOC individuals was nearly three times larger compared with controls (Table 1). In contrast, novel duplications in HBOC individuals were observed less frequently compared with controls (Table 1).

In this study, we focused on CNVs with the following characteristics: they were enriched in HBOC individuals compared with controls and 1) affected known or potential genes contributing to HBOC predisposition (3 CNVs); or 2) they were homozygous, and carriers presented with interesting clinical outcomes (1 CNV); or 3) they were not reported in the Database of Genomic Variants and affected genes related to BC (2 CNVs). CNVs of interest were confirmed by another program (QuantiSNP or cnvPartition). In total, six CNVs were selected for further validation by qPCR, and they were genotyped in additional cohort of index individuals from 20 HBOC families and five of the CNVs were genotyped in 299–869 additional healthy female controls (Table 2). The CNVs were correlated with clinical data from the HBOC individuals (Table 3).

**Table 2:** Validated copy number variations.

				Carrier frequency				
Cytoband^a^	Gene(s)	Type	Size (kb)^b^	HBOC ind^c^	Controls^d^	*P*-values	OR; 95%CI	Status^e^
2q34	*ERBB4*	intronic deletion	28.7–59.0	0.050 (5/101)	0.034 (12/358)	0.457	1.49; 0.52–4.28	Novel
3p11.1	*EPHA3*	intronic deletion	14.6	0.119 (12/101)	0.063 (27/432)	0.055	1.96; 0.97–3.94	Reported
5q15	*–*	intergenic deletion	49.8	0.050 (5/101)^f^	0.063 (57/899)	0.845	0.92; 0.39–2.16	Reported
8p23.2	*CSMD1*	intronic deletion	10.8	0.010 (1/101)	0.002 (1/436)	0.259	4.33; 0.27–69.57	Novel
17q21.31	*BRCA1, NBR1, NBR2*	exonic deletion	99.0	0.010 (1/101)	0 (0/35)	0.555	na	Reported
19q13.41	*ERVV-2*	exonic duplication	15.8–26.9	0.109 (11/101)^g^	0.102 (34/334)	0.322	1.37; 0.73–2.55	Reported

Abbreviations: CI = confidence interval; na = not available; OR = odds ratio.

a According to the NCBI Genome Build 36.1 (hg 18). Exact start and end positions of the CNVs are provided in Table S1.

b Size reported in HBOC individuals analysed in the SNP array (may vary between individuals).

c Combined frequencies of original cohort of 81 HBOC individuals (analysed in the SNP array) and cohort of 20 additional HBOC individuals (genotyped by TaqMan® Copy Number Assays). CNVs in the 2q34, 5q15, 8p23.2, and 17q21.31 regions were not observed in additional cohort of 20 HBOC individuals. Heterozygous deletion (copy number 1) in the 3p11.1 region was also identified in 4 out of the 20 additional HBOC individuals (File S2). Heterozygous duplication (copy number 3) in the 19q13.41 region was also identified in 3 out of the 20 additional HBOC individuals (File S2). Homozygous duplication (copy number 4) in the 19q13.41 region was identified in 1 out of the 20 additional HBOC individuals (File S2).

d Thirty-five controls were first analyzed in the SNP array. CNVs were also screened in additional controls by TaqMan® Copy Number Assays (excluding *BRCA1* affecting CNV since large deletions in *BRCA1* coding regions are known to associate with breast and ovarian cancer susceptibility).

e Search against the Database of Genomic Variants (DGV).

f Deletion in the 5q15 region was homozygous (copy number 0) in 1 out of the 101 (0.010) HBOC individuals and in 1 out of the 899 (0.001) controls and heterozygous (copy number 1) in 4 out of the 101 (0.040) HBOC individuals and in 56 out of the 899 (0.062) controls.

g Duplication in the 19q13.41 region was homozygous (copy number 4) in 4 out of the 101 (0.040) HBOC individuals and in 3 out of the 334 (0.009) controls and heterozygous (copy number 3) in 7 out of the 101 (0.069) HBOC individuals and in 31 out of the 334 (0.093) controls.

**Table 3:** The clinical characteristics and family cancer history for HBOC individuals analysed in the SNP array with the six validated copy number variations.

Family	Variation	Cancer (age at dg)	Br/Ov Cahistology/grade	Receptor Status	Ca cases in the family(age at dg if known)
221	2q34 del	Bil. Br (39, 42)	duct, gr 1 and	ER+, PR+, HER2− and	Br (51), *Panc (54)*
			duct, gr 2	ER+, PR+, HER2−	
212	2q34 del	Bil. Br (43)	duct, gr na and na	ER+, PR+, HER2− and na	Br (52)
263	2q34 del	Ov (69), Br (72)	duct, gr 3	ER−, PR−, HER2−	–
249	2q34 del	Br (42)	medullary, na	na	Br (*35*, **44,** 57, 67, 71), Ute (39), Kid (67), Mel (63)
					*Ov (45)*, Skin, To (51), Co (78)
132	2q34 del	Br (47)	duct, gr 1	ER+, PR+, HER2 na	Br (38)
232	3p11.1 del	Br (34)	duct, na	ER+, PR+, HER2 na	Br (39)
244	3p11.1 del	Br (45)	duct, gr 2	ER+, PR+, HER2−	Bil. Br (<45), Br (<35, 46), Brain (67)
121	3p11.1 del	Br (50)	duct, gr 3	ER−, PR−, HER2+	4xBr (36, 39, 40, 48)
207	3p11.1 del	Br (38)	duct, gr 3	na	Bil.Br (64)
230	3p11.1 del	Br (33), Kid (37)	duct, gr 1	ER+, PR+, HER2−	*Br (70)*
118	3p11.1 del	Ov (32), Br (40), Mel (41)	Mucinous and	ER+, PR+, HER2−	–
	19q13.41 dup		duct, gr 2		
269	3p11.1 del	Br (36)	duct, gr 1	ER+, PR+, HER2−	*3xBr (52, 70, 72), Skin (66)*
225	3p11.1 del	Br (43)	duct, gr 1	ER+, PR+, HER2−	2xBr (52, 77), *Kid (64)*
123	5q15 del*	Br (29)	duct, gr 2	ER+, PR−, HER2−	Br (65), Eso (73)
250	5q15 del	Br (24)	duct, gr 3	ER+, PR+, HER2+	Cer (30), *Ov (83)*
264	5q15 del	Bil. Br (44)	lob, gr 2	ER+, PR+, HER2−	Br (44, *52*)
	19q13.41 dup				
129	5q15 del	BCC (70), Bil. Br (78),	left: lob, gr 2,	left: ER−, PR−, HER2−,	Bil. Br (59), **BCC (48), Co (58)**
		Sto (82)	right: duct, gr 1	right: ER+, PR+, HER2−	
246	5q15 del	Thy (30), Cer (33), Br (39)	duct, gr 3	ER−, PR−, HER2+	2xBr (**49**, 54), *Rectum (61)*
128	8p23.2 del	Br (36)	duct, gr 2	ER+, PR+, HER2−	*2x Br (45, 58)*, *GI (57),* Mel (69)
252	17q21.31 del	Br (46)	duct, gr 3	ER−, PR−, HER2−	Bil. Ov (46), Ov (44)
240	19q13.41 dup*	Br (53)	duct, gr 3	ER+, PR−, HER2+	2xBr (42, 62)
206	19q13.41 dup	Br (53)	duct, gr 1	ER−, PR−, HER2−	Bil. Br (64), **Br (49)**
133	19q13.41 dup	Br (48)	duct, gr 2	ER+, PR+, HER2−	*2xBr (73, 79)*, *Int*, BCC (60)
113	19q13.41 dup*	Br (51), BCC (55)	duct, gr 3	ER−, PR−, HER2+	Br (35)
239	19q13.41 dup*	Br (37)	duct, gr 2	ER+, PR+, HER2−	*Br (>90), Co*

* Homozygous CNV.

Abbreviations: BCC = Basal-cell carsinoma; Bil. Br = bilateral breast; Br = breast; Ca = cancer; Cer = cervix in situ carsinoma/cervix carsinoma; Co = colon; Dg = diagnosis; Del = deletion; Duct = ductal; Dup = duplication; Eso = esophagus; GI = gastrointestinal; gr = grade; Int = intestine; Kid = kidney; Lob = lobular; Mel = melanoma; na = not available; Ov = ovary; Panc = pancreatic; Sto = stomach; Thy = thyroid; To = tongue; Ute = ute. Cancers diagnosed in the paternal side of the family are presented in italics. Cancers diagnosed in siblings or their children of the index patients are underlined. Cancers diagnosed in the children of the index patients are presented in bold.

All six validated CNVs listed in Table 2 are located in genomic regions related to BC. CNVs in the intronic regions of *ERBB4* and *EPHA3* were enriched in HBOC individuals compared with controls (Table 2). *EPHA3* and *ERBB4* encode proteins that are involved in important signalling pathways. A homozygous deletion in the 5q15 locus was identified in one BC patient (1 out of the 101, 1.0%) with drastic clinical characteristics (Tables 2 and 3). This homozygous deletion was observed only in 1 out of the 899 (0.1%) healthy controls (Table 2). Deletions affecting the intronic region of the *CSMD1* tumor suppressor gene and exonic regions of the highly penetrant *BRCA1* were observed only in 1 out of the 101 (1.0%) HBOC individuals and *CSMD1* deletion was identified in 1 out of the 436 (0.2%) controls (Table 2). Because large deletions in *BRCA1* are known to predispose to HBOC, there was no need to screen for the deletion in additional controls. A duplication affecting the coding region of the *ERVV-2* gene, which belongs to endogenous retroviruses, was more commonly homozygous in HBOC individuals compared with controls (Table 2).

**Figure 1:**
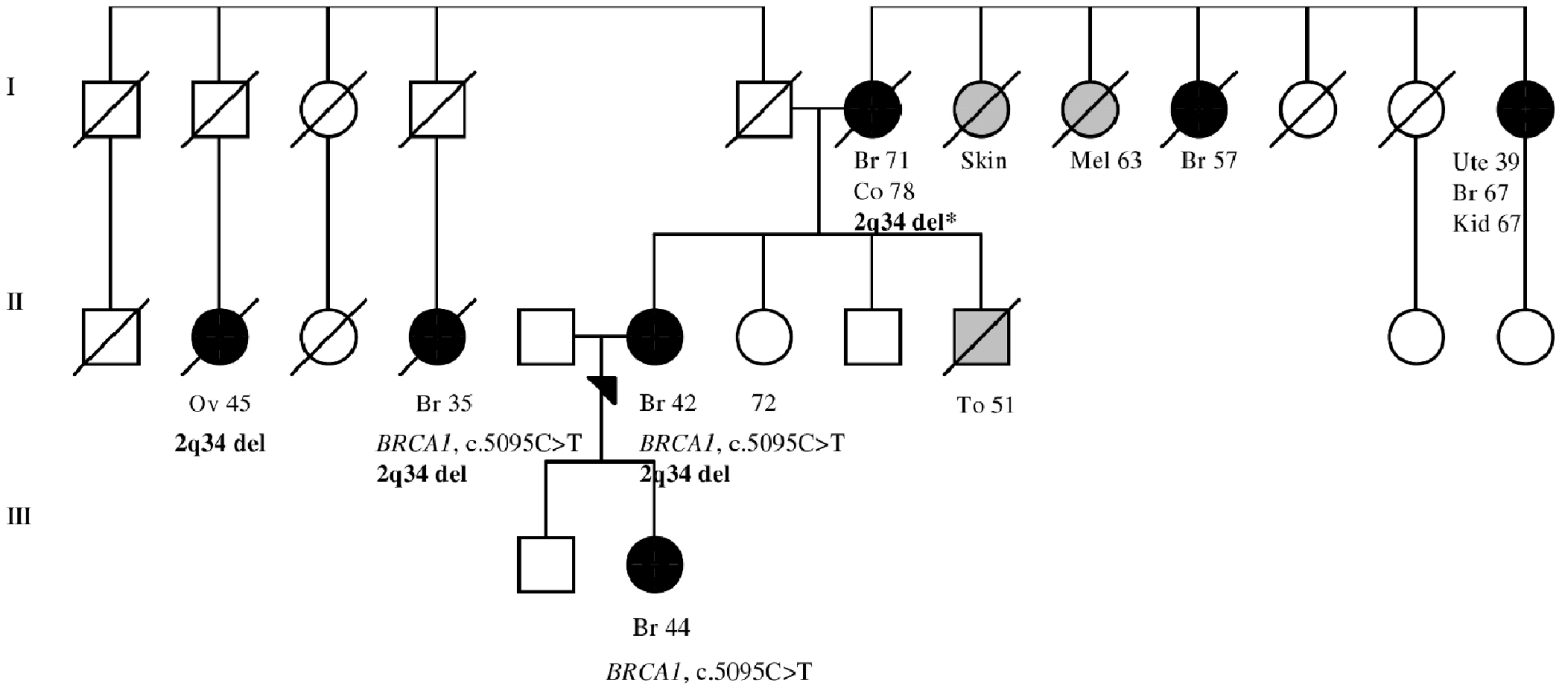
Family 249 pedigree. Index individual carries a novel 59.0 kb deletion in the 2q34 locus. The deletion affects intronic region of the *ERBB4* gene, which encodes a receptor tyrosine kinase family member that plays an important role in several cellular signalling pathways. The deletion was also identified in index’s mother and two paternal cousins. Mother carried homozygous deletion (indicated with an asterisk). Index’s daughter was tested to be negative for the deletion. Additionally, deleterious *BRCA1* c.5095C>T variant has been previously identified in three individuals in the family. Females are marked with circles and males are marked with squares. Index individual is marked with an arrow. Breast and ovarian cancers are marked with black circles with the age at diagnosis. Other cancers are marked with grey and specified with the age at diagnosis (Br: breast, Co: colon, Kid: kidney, Mel: melanoma, Ov: ovarian, To: tongue, Ute: uterus). Deceased individuals are marked with a slash. Current age of index’s healthy sister is indicated. Generations are marked with the Roman numerals on the left. The pedigree figure has been modified from Kuusisto *et al*, 2011 [18].

**Figure 2:**
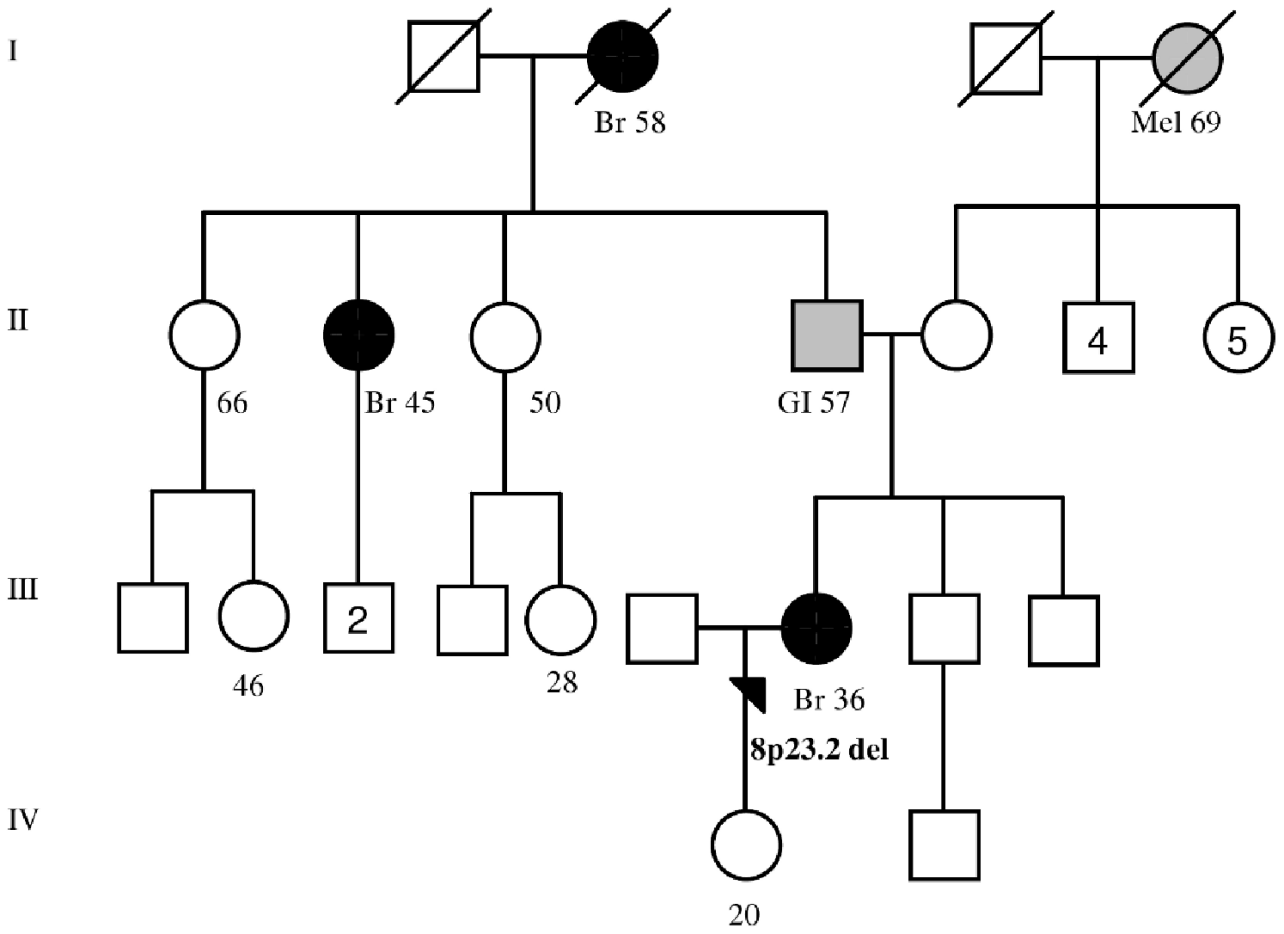
Family 128 pedigree. Index individual carries a novel 10.8 kb deletion in the 8p23.2. The deletion affects intronic region of the *CSMD1* tumor suppressor gene. Females are marked with circles and males are marked with squares. Number in circle or squares indicates descendants. Index individual is marked with an arrow. Breast cancers are marked with black circles with the age at diagnosis. Other cancers are marked with grey and specified with the age at diagnosis (Br: breast, GI: gastrointestinal, Mel: melanoma). Deceased individuals are marked with a slash. Current ages of healthy females are presented in the paternal side of the family. In addition, the current age of index’s healthy daughter is indicated. Generations are marked with the Roman numerals on the left.

The clinical characteristics and family cancer history for individuals with HBOC with the six validated CNVs are presented in Table 3 (only CNVs identified in our original cohort of 81 HBOC individuals first analysed in the SNP array are presented). Most importantly, 2 out of the 5 individuals with HBOC with a novel deletion at the 2q34 *ERBB4* locus had bilateral BC that was diagnosed at ≤43 years of age (Table 3; families 221 and 212). We were able to analyse the segregation of the 2q34 deletion in family 249 (Table 3) in which a deleterious *BRCA1* variant was previously identified in three individuals (Figure 1) [18]. The 2q34 deletion was identified in the index’s mother (homozygous) and two paternal cousins (heterozygous) (Figure 1). However, the index’s daughter did not carry the deletion (Figure 1). A common feature for all of the 3p11.1 deletion (at *EPHA3* locus) carriers was ductal BC diagnosed at ≤50 years and positive hormone receptor status (6 out of the 8 carriers) in the cohort of 81 HBOC individuals (Table 3). In the second cohort of 20 additional HBOC individuals, 3p11.1 deletion was identified in two BC patients, one ovarian cancer patient and a patient who had both breast and ovarian cancer (File S2). Interestingly, all three patients with BC presented ductal form of the cancer and estrogen and progesterone receptor positive status (File S2). Intergenic deletion in the 5q15 region was of great interest because it was found as a homozygous deletion in a BC patient who was diagnosed at age 29 years and died of BC at the same age (Table 3, family 123). Additionally, one heterozygous 5q15 deletion carrier had BC diagnosed at an early age (24 years) and the other had thyroid and cervical cancers in addition to BC diagnosed before age 40 years (Table 3; families 250 and 246). A novel deletion of high interest at 8p23.2, which affects the *CSMD1* intronic region, was identified in a patient with ductal grade 2, hormone receptor positive BC diagnosed at a relatively early age (36 years) with a paternal family history of BC (Table 3, family 128 and Figure 2). A deletion affecting *BRCA1, NBR1,* and *NBR2* at 17q21.31 was identified in a patient with hormone receptor-negative BC with a family history of ovarian cancer (Table 3, family 252).

Enrichment analysis was performed for the CNV-affecting genes to identify common functions of the gene products. *EPHA3, ERBB4* and *BRCA1* were identified in several GO term categories and pathways that were significantly overrepresented (P<0.05) (presented in detail in Table S2). Both *EPHA3* and *ERBB4* were identified to have molecular functions related to receptor activity, transmembrane receptor activity, molecular transducer activity, and signal transducer activity. In contrast, *BRCA1* was identified in several pathways related to DNA double-strand breaks and repair. In addition, Online Mendelian Inheritance in Man (OMIM) and The Genetic Association database searches revealed the role of *CSMD1* in BC.

## Discussion

In this study, we aimed to identify CNVs contributing to HBOC susceptibility in Finland and obtain new information for the genetic counselling of HBOC families. We utilised a cohort of well-characterised *BRCA1/2*-founder mutation-negative individuals from 84 Finnish hereditary breast and/or ovarian cancer families who had been previously screened for variations in seven known BC genes [18].

Here, we identified more gene-disrupting deletions in HBOC individuals compared with controls suggesting that altered function of their protein products, particularly in critical pathways, could explain pathogenic events in HBOC individuals. Additionally, a proportion of novel gene-affecting deletions, which were not reported in healthy controls in the database, was higher in HBOC individuals compared with controls, suggesting that these novel CNVs are more likely to be disease -related.

We focused on CNVs that were enriched in HBOC individuals compared with controls and affected genes that likely play a role in HBOC predisposition. In addition, one intergenic deletion was also included for further validation based on the homozygous form of the aberration and notably poor clinical characteristics of the carrier. Thus, six CNVs were considered to be the most relevant for further validation. Because our sample number in the SNP array was limited, we also genotyped the six CNVs in a cohort of 20 additional HBOC individuals. Furthermore, five of the CNVs were genotyped in 299–869 additional healthy controls. Because clinical characteristics of the additional cohort of 20 HBOC individuals were comparable to our original cohort of 81 HBOC individuals, we combined the observed frequencies of the CNVs in both cohorts in Table 2. Additionally, we performed segregation analysis of one family to determine how the CNV co-segregated with the disease and another BC-associated variant. The CNVs were compared with the clinical data of the HBOC individuals.

In this study, the most frequently observed aberration in HBOC individuals was a deletion disrupting the *EPHA3* intronic region (Table 2). *EPHA3* belongs to the ephrin receptor subfamily of the receptor tyrosine kinase (RTK) family, which plays an important role in normal cell physiology and disease pathogenesis [24]. Ephrin receptor signalling together with ephrin-ligands is known to regulate both tumour growth and suppression in several different cancers including BC [25]. According to recent studies, altered *EPHA3* expression is associated with gastric and colorectal cancers, and CNVs in the *EPHA3* region have been found to be associated with haematologic malignancies [26]–[28]. However, haematologic malignancies were not observed in *EPHA3* deletion carriers in this study. Our data suggest that an intronic deletion may disrupt the *EPHA3* regulatory elements, thus leading to altered protein function and pathogenic BC events. Thus, considering the important role of *EPHA3* in signalling pathways, the segregation of the intronic deletion should be studied in the families and the deletion should be further screened in a larger sample set.

The intergenic 5q15 deletion, particularly as a homozygous deletion, is highly interesting from a clinical perspective. This deletion was identified in a patient who had been diagnosed with BC at age 29 and died of the disease at the same age. Homozygous deletion of the 5q15 locus was extremely rare in healthy controls (1 out of the 899, 0.1%) (Table 2), which emphasises the importance of the variation. Moreover, it is possible that a fraction of the anonymous controls may develop breast or ovarian cancer later in life although they were healthy at the time of the blood draw. The 5q15 deletion may affect the transcriptional control of target gene expression. Regulatory elements of the target gene can extend to long distances outside of the transcription unit [29], which makes gene expression regulation a complex process. Interestingly, aberrant expression of the nearest neighbouring gene (1.0 Mb distance), *RGMB*, has been implicated in BC [30]. Additional analysis is needed to determine whether *RGMB* regulatory elements exist in the 5q15 deletion locus. Moreover, a previous copy number study of breast tumours-associated aberrations in the 5q15–5q21 locus with p53 status and patient survival suggests that the 5q15 region may be important in BC predisposition [31]. Furthermore, to reveal possible functional elements located in the deletion region, the Encyclopedia of DNA Elements (ENCODE) (http://genome.ucsc.edu/ENCODE/) was utilised. Preliminary analysis revealed enhancer and promotor-associated histone mark (H3K4Me1) activity and DNase hypersensitivity, which indicate that regulatory elements are active in this genomic region. Thus, the 5q15 homozygous deletion requires special attention because it may have clinical significance for screening families with BC with early disease onset. Interestingly, two heterozygous 5q15 loss carriers with lobular BC (Table 3, families 264 and 129) were previously found to carry BC-associated *CHEK2* variants [18].

The novel 8p23.2 deletion affects an intronic region in the *CSMD1* tumour suppressor gene. *CSMD1* has mainly been associated with head and neck squamous cell carcinoma, but CSMD1 losses is also reported to contribute to the tumourigenesis of several other epithelial cancers, including BC [32]. In addition, *CSMD1* deletions and aberrant splicing have been shown to contribute to altered CSMD1 function in vivo [32]. Moreover, decreased CSMD1 expression has been associated with high tumour grade and the poor survival of invasive ductal breast carcinoma, and the role of CSMD1 expression as a potential BC prognostic marker has been suggested [33]. In this study, the *CSMD1*-affecting intronic deletion was identified in the index individual for one BC family (1 out of the 101, 1.0%) (family 128, Figure 2 and Table 3). In this family, the index patient and her paternal aunt and grandmother had been diagnosed with BC at ages 36, 45, and 58 years, respectively (Figure 2). In addition, gastrointestinal cancer was diagnosed on the paternal side of the family (father) (Figure 2). Interestingly, the *CSMD1*-affecting deletion was observed only in 1 out of the 436 (0.2%) healthy controls, suggesting that this rare variant likely predisposes individuals to BC. We are currently seeking DNA samples from the other family members (family 128) to determine whether the variation co-segregates with BC in the family. In addition, although the deletion should be screened for in larger sample set, the *CSMD1* gene is a potential candidate for the further study of HBOC susceptibility in Finnish families.

A novel deletion at 2q34 affects the intronic region of the *ERBB4* gene, which is known to play a role in BC [34]. *ERBB4* encodes an epidermal growth factor RTK subfamily member that regulates several cellular processes and plays an important role in cancer [35]. We found that the aberration in *ERBB4* is 1.5 times more common in HBOC individuals compared with controls suggesting that it may be a disease-related low-risk variant (Table 2). In addition, the clinical features of the *ERBB4* deletion carriers were interesting because two of the HBOC individuals had bilateral BC diagnosed at a relatively early age (Table 3). To further analyse the deletion, we were able to perform a segregation analysis in one family in which a deleterious *BRCA1* c.5095C>T variant was previously recognised (Figure 1) [18]. Thus, three BC cases in the family (index, index’s daughter and paternal cousin) are explained by the paternally inherited high-penetrant *BRCA1* variant. The *ERBB4* deletion was observed on the maternal and paternal sides of the family (Figure 1). However, in the mother, who had BC diagnosed at an older age, the *ERBB4* deletion was homozygous, suggesting that the deletion could contribute to BC development at an older age, particularly in its homozygous form. Thus, it would be interesting to screen for the deletion in other BC cases diagnosed at an older age on the mother’s side of the family as well. Additionally, an ovarian cancer patient who was negative for the highly -penetrant *BRCA1* variant was found to carry a heterozygous form of the 2q34 deletion, suggesting that the deletion may also contribute to ovarian cancer risk to some extent (Figure 1).

*BRCA1* deletions are known to predispose to breast/ovarian cancer [36]. In this study, a large deletion overlapping exons 1A-13 of *BRCA1* was observed in one individual with BC diagnosed at age 46 years and with ovarian cancers diagnosed in her mother and half-sister (Table 3, family 252). In our previous analysis, the sample was excluded from the MLPA analysis due to a low sample quality value [18]. The *BRCA1* deletion encompassing exons 1A-13 has been reported in a Finnish breast/ovarian cancer family [37]. Here, the deletion was found to affect also the neighbouring genes *NBR1* (entire gene) and *NBR2* (exons 1–10) according to the PennCNV, QuantiSNP and cnvPartition programs. Similar findings have been reported worldwide in a few studies [38], [39]. Because the *BRCA1* deletion is known to be clinically relevant, MLPA analysis was performed to validate the *BRCA1* deletion (Figure S1). Genetic counselling was offered for the deletion carrier patient.

The duplication identified at 19q13.41 affects exon 1 of the *ERVV-2* gene. *ERVV-2* belongs to the human endogenous retrovirus (ERV) family and the involvement of ERVs in the pathogenesis of human cancer has been suggested but their roles in biological disease processes are poorly understood [40]. Because 19q13 genomic region has been previously associated with BC [41], this prompted us to further examine the duplication affecting the *ERVV-2* coding region. Screening for the duplication in additional controls revealed that it was as common in controls compared with HBOC individuals (Table 2). However, the homozygous form of the variation was 4.4 times more common in HBOC individuals compared with controls (Table 2), suggesting that the aberration may contribute to breast and ovarian cancer risk to some extent, but further studies are needed to confirm the findings. Of interest, one of the homozygous duplication carriers (Table 3, family 240) had been reported to carry a novel *BRCA2* variant predicted to be pathogenic [18].

In conclusion, this study is a continuation of our previous work with the aim of elucidating genetic factors contributing to HBOC susceptibility in Finland. We have identified several potential CNVs that likely increase the risk of HBOC susceptibility that may thus explain a fraction of breast and ovarian cancer cases. The aberrations at 3p11.1, 5q15, and 8p23.2 regions require special attention because they may be utilised for the genetic counselling of HBOC families, but more studies are needed to confirm the preliminary findings.

## Supporting Information

Figure S1 ***BRCA1*** **deletion (exons 1A-13) confirmation by MLPA.**
(PDF)

Table S1 **All of the identified 545 copy number variations (CNVs) at 273 different genomic regions (listed according to** ***P*** **-values).**
(PDF)

Table S2 **Enriched GO term categories and pathways (** ***P*** **-value less than 0.05) involving** ***EPHA3*** **,** ***ERBB4*** **and** ***BRCA1.***
(PDF)

File S1 **Clinical characteristics of three additional individuals.**
(PDF)

File S2 **Clinical characteristics of 20 additional HBOC individuals utilised for CNV validation analysis.**
(PDF)

File S3 **Copy number variation validation protocol by quantitative RT-PCR.**
(PDF)
