# Supplementary material for: Copy Number Variation Analysis in Familial BRCA1/2-Negative Finnish Breast and Ovarian Cancer
Source: PLoS One. 2013 Aug 13;8(8):e71802. doi: 10.1371/journal.pone.0071802 (PMC3742470; doi:10.1371/journal.pone.0071802)
Supplement: Figure S1 — BRCA1 deletion (exons 1A-13) confirmation by MLPA. (PDF) [file pone.0071802.s001.pdf]

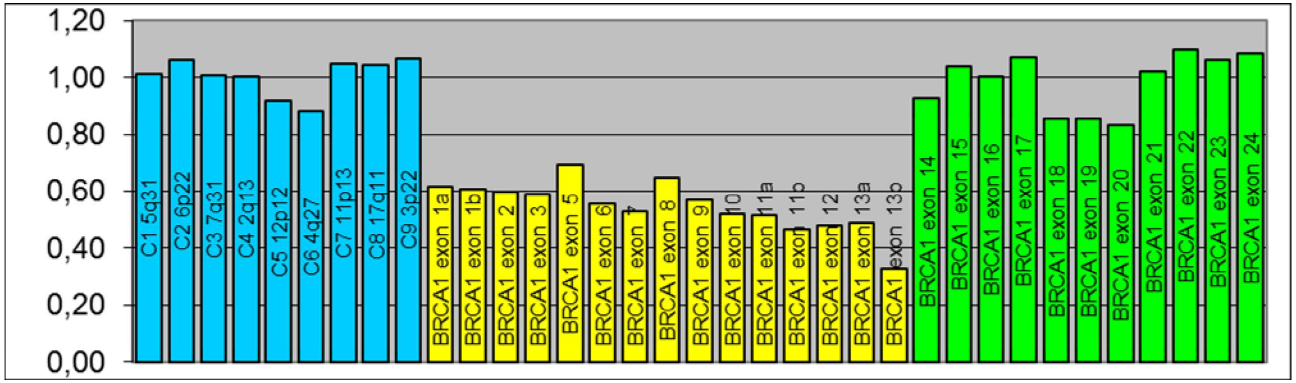

Figure S1. *BRCA1* deletion (exons 1A-13) confirmation by MLPA. *BRCA1* deletion (exons 1A-13) was confirmed in one breast cancer patient with family history of ovarian cancer (index patient for family 252). MLPA analysis was performed for *BRCA1* by SALSA MLPA POO2 *BRCA1* probemix and SALSA MLPA EK1 reagent kit (lot C2-0811, MRC-Holland, Amsterdam, the Netherlands) according to manufacturer's instructions and analyzed with ABIPRISM 3130xl Genetic Analyzer (Applied Biosystems, Foster City, CA, USA). The data was first visualised with Peak Scanner Software v1.0 (Applied Biosystems) and analysed with the National Genetics Reference Laboratory (Manchester, UK) Spreadsheet according to the manufacturer's instructions. Control probes are marked with blue. Deletion affected probes are marked with yellow. Probes, which are not affected by the deletion, are marked with green. As deletion control, DNA sample with *BRCA1* exon 13 deletion was used [1].

References

1. Laurila E, Syrjakoski K, Holli K, Kallioniemi A, Karhu R. (2005) Search for large genomic alterations of the BRCA1 gene in a finnish population. Cancer Genet Cytogenet 163: 57-61.
