# Supplementary material for: Copy Number Variation Analysis in Familial BRCA1/2-Negative Finnish Breast and Ovarian Cancer
Source: PLoS One. 2013 Aug 13;8(8):e71802. doi: 10.1371/journal.pone.0071802 (PMC3742470; doi:10.1371/journal.pone.0071802)
Supplement: Table S1 — All of the identified 545 copy number variations (CNVs) at 273 different genomic regions (listed according to P -values). (PDF) [file pone.0071802.s002.pdf]

Table S1. All of the identified 545 copy number variations (CNVs) at 273 different genomic regions (listed according to *P*-values).

						No of CNVs in				
**Cytoband**	**Chromosomal region[a]**	**Size (kb)**	**Type**	**Gene**	**Exon**	**HBOC ind**	**Controls**	***P*-value**	**OR**	**95%CI**
3p11.1	chr3:89485137-89499754	14618	Deletion	*EPHA3*	*intronic*	8	0	0,051	Inf	na
19q13.41	chr19:58213912-58240762	26851	Duplication	*ERVV-2*	*ERVV-2*	7	0	0,075	Inf	na
15q11.2	chr15:19788495-19962000	173506	Deletion	*LOC727924,OR4M2, OR4N3P,OR4N4*	*LOC727924,OR4M2, OR4N3P,OR4N4*	6	0	0,109	Inf	na
5q15	chr5:97075236-97125076	49841	Deletion	*intergenic*	*intergenic*	5	0	0,160	Inf	na
22q13.1	chr22:37695722-37715456	19735	Deletion	*APOBEC3A,APOBEC3B*	*APOBEC3B*	5	0	0,160	Inf	na
1p21.1	chr1:102471625-102616313	144689	Deletion	*MIR548AI*	*MIR548AI*	4	0	0,232	Inf	na
2q35	chr2:215221675-215254549	32875	Deletion	*intergenic*	*intergenic*	4	0	0,232	Inf	na
7q11.23	chr7:76325606-76453285	127680	Duplication	*PMS2P11*	*PMS2P11*	4	0	0,232	Inf	na
11p11.12	chr11:51048278-51051138	2861	Deletion	*intergenic*	*intergenic*	11	3	0,337	1,67	na
14q11.2	chr14:19283777-19490411	206635	Duplication	*OR4K1,OR4K2, OR4K5,OR4M1, OR4N2,OR4Q3*	*OR4K1,OR4K2, OR4K5,OR4M1, OR4N2,OR4Q3*	8	2	0,370	1,80	na
13q31.1	chr13:83009774-83045171	35398	Deletion	*intergenic*	*intergenic*	10	3	0,406	1,50	na
12p11.21	chr12:31160567-31292645	132079	Duplication	*intergenic*	*intergenic*	5	1	0,412	2,22	na
19p12	chr19:20423788-20507201	83414	Deletion	*intergenic*	*intergenic*	7	2	0,453	1,56	na
1p36.33	chr1:1610720-1661642	50923	Deletion	*CDK11A,CDK11B, MMP23A,SLC35E2, SLC35E2B*	*CDK11A,CDK11B, MMP23A,SLC35E2, SLC35E2B*	2	0	0,486	Inf	na
1p36.22	chr1:12400493-12438178	37686	Duplication	*VPS13D*	*intronic*	2	0	0,486	Inf	na
1p36.22-36.21	chr1:12496410-12615712	119303	Duplication	*DHRS3*	*DHRS3*	2	0	0,486	Inf	na
1p36.21	chr1:12698721-12838434	139714	Duplication	*AADACL3,C1orf158, HNRNPCL1,LOC649330,*	*AADACL3,C1orf158, HNRNPCL1,LOC649330,*	2	0	0,486	Inf	na na

1p36.21	chr1:12789486-12874361	84876	Deletion	*PRAMEF1,PRAMEF11, PRAMEF12 HNRNPCL1,LOC649330, PRAMEF11,PRAMEF2, PRAMEF4*	*PRAMEF1,PRAMEF11, PRAMEF12 HNRNPCL1,LOC649330, PRAMEF11,PRAMEF2, PRAMEF4*	2	0	0,486	Inf	na
1p33	chr1:49691764-49762922	71159	Deletion	*AGBL4*	*intronic*	2	0	0,486	Inf	na
2p16.3	chr2:51496826-51545910	49085	Deletion	*intergenic*	*intergenic*	2	0	0,486	Inf	na
6p12.1	chr6:55939140-55954197	15058	Deletion	*intergenic*	*intergenic*	2	0	0,486	Inf	na
8q21.13	chr8:83435642-83454850	19209	Deletion	*intergenic*	*intergenic*	2	0	0,486	Inf	na
9p24.3	chr9:196132-234457	38326	Duplication	*C9orf66,DOCK8*	*C9orf66,DOCK8*	2	0	0,486	Inf	na
10q21.3	chr10:67748487-67784487	36001	Deletion	*CTNNA3*	*intronic*	2	0	0,486	Inf	na
14q11.2	chr14:21845319-21864966	19648	Duplication	*intergenic*	*intergenic*	2	0	0,486	Inf	na
15q11.2	chr15:21606581-21612590	6010	Deletion	*intergenic*	*intergenic*	2	0	0,486	Inf	na
15q11.2	chr15:22134751-22320561	185811	Duplication	*intergenic*	*intergenic*	2	0	0,486	Inf	na
19q13.31	chr19:48064226-48157011	92786	Duplication	*PSG1,PSG6,PSG7*	*PSG1,PSG6,PSG7*	2	0	0,486	Inf	na
1q21.1	chr1:147305744-147478120	172377	Duplication	*intergenic*	*intergenic*	4	1	0,523	1,76	na
2q34	chr2:212065237-212124274	59038	Deletion	*ERBB4*	*intronic*	5	1	0,523	1,76	na
3q26.1	chr3:163617384-163625169	7786	Deletion	*intergenic*	*intergenic*	4	1	0,523	1,76	na
1q21.1	chr1:147305744-147478120	172377	Deletion	*intergenic*	*intergenic*	15	6	0,543	1,10	na
7p22.3	chr7:141322-162448	21127	Duplication	*intergenic*	*intergenic*	3	1	0,649	1,30	na
11p11.12	chr11:49671841-49709533	37693	Deletion	*LOC440040*	*intronic*	3	1	0,649	1,30	na
12p13.31	chr12:7891603-8014573	122971	Duplication	*SLC2A14,SLC2A3*	*SLC2A14,SLC2A3*	3	1	0,649	1,30	na
17p11.2	chr17:19443475-19478062	34588	Deletion	*intergenic*	*intergenic*	3	1	0,649	1,30	na
10q11.22	chr10:47063139-47171576	108438	Duplication	*ANTXRL*	*ANTXRL*	9	4	0,655	0,97	na
1p36.22	chr1:12341287-12373416	32130	Duplication	*VPS13D*	*VPS13D*	1	0	0,698	Inf	na
1p36.11	chr1:25470863-25515044	44182	Deletion	*RHD*	*RHD*	1	0	0,698	Inf	na
1p33	chr1:49900785-49964737	63953	Deletion	*AGBL4*	*AGBL4*	1	0	0,698	Inf	na
1p22.1	chr1:94402231-94461615	59385	Deletion	*ARHGAP29*	*ARHGAP29*	1	0	0,698	Inf	na

1q31.3	chr1:192902103-192948685	46583	Duplication	*intergenic*	*intergenic*	1	0	0,698	Inf	na
1q41	chr1:216085579-216121310	35732	Deletion	*SPATA17*	*SPATA17*	1	0	0,698	Inf	na
1q42.3	chr1:234424014-234500747	76734	Deletion	*ERO1LB,GPR137B*	*ERO1LB,GPR137B*	1	0	0,698	Inf	na
2p21	chr2:47443538-47460278	16741	Deletion	*EPCAM,MIR559*	*EPCAM,MIR559*	1	0	0,698	Inf	na
2p16.3	chr2:49534201-49592865	58665	Deletion	*intergenic*	*intergenic*	1	0	0,698	Inf	na
2p16.2	chr2:53256546-53293133	36588	Deletion	*intergenic*	*intergenic*	1	0	0,698	Inf	na
2p12	chr2:82899312-82952000	52689	Deletion	*LOC1720*	*LOC1720*	1	0	0,698	Inf	na
2p11.2	chr2:87482067-87633978	151912	Deletion	*NCRNA00152*	*NCRNA00152*	1	0	0,698	Inf	na
2q14.1	chr2:113811749-114209785	398037	Duplication	*CBWD2,DDX11L2, FAM138B,FOXD4L1, MIR4782,RABL2A, RPL23AP7,SLC35F5, WASH2P*	*CBWD2,DDX11L2, FAM138B,FOXD4L1, MIR4782,RABL2A, RPL23AP7,SLC35F5, WASH2P*	1	0	0,698	Inf	na
2q14.3	chr2:123502132-123526178	24047	Duplication	*intergenic*	*intergenic*	1	0	0,698	Inf	na
2q14.3	chr2:123986255-124017655	31401	Deletion	*intergenic*	*intergenic*	1	0	0,698	Inf	na
2q23.1	chr2:148968172-149060899	92728	Duplication	*MBD5*	*MBD5*	1	0	0,698	Inf	na
2q24.3	chr2:168227203-168311130	83928	Duplication	*intergenic*	*intergenic*	1	0	0,698	Inf	na
2q31.3	chr2:181176255-181179083	2829	Duplication	*intergenic*	*intergenic*	1	0	0,698	Inf	na
2q33.1	chr2:197174006-197209016	35011	Duplication	*intergenic*	*intergenic*	1	0	0,698	Inf	na
2q33.2	chr2:204163416-204245415	82000	Duplication	*intergenic*	*intergenic*	1	0	0,698	Inf	na
2q33.2	chr2:204554473-204920874	366402	Duplication	*intergenic*	*intergenic*	1	0	0,698	Inf	na
2q34	chr2:212404169-212421080	16912	Duplication	*ERBB4*	*intronic*	1	0	0,698	Inf	na
2q36.3	chr2:227941797-227954773	12977	Duplication	*TM4SF20*	*TM4SF20*	1	0	0,698	Inf	na
2q37.1	chr2:232923704-233017792	94089	Duplication	*ALPP,ALPPL2, ECEL1P2*	*ALPP,ALPPL2, ECEL1P2*	1	0	0,698	Inf	na
3p26.2	chr3:5375168-5407114	31947	Deletion	*intergenic*	*intergenic*	1	0	0,698	Inf	na
3p12.3	chr3:79268774-79482170	213397	Deletion	*ROBO1*	*intronic*	1	0	0,698	Inf	na
3q11.2	chr3:95114888-95127737	12850	Duplication	*PROS1*	*PROS1*	1	0	0,698	Inf	na

4p16.3	chr4:75800-101487	25688	Duplication	*ZNF595,ZNF718*	*ZNF595*	1	0	0,698	Inf	na
4p16.3	chr4:1930398-1939679	9282	Duplication	*WHSC1*	*WHSC1*	1	0	0,698	Inf	na
4p16.1	chr4:9855546-10028563	173018	Duplication	*intergenic*	*intergenic*	1	0	0,698	Inf	na
4p16.1	chr4:10156441-10476154	319714	Duplication	*CLNK*	*CLNK*	1	0	0,698	Inf	na
4p16.1	chr4:10568873-10700494	131622	Duplication	*intergenic*	*intergenic*	1	0	0,698	Inf	na
4q12	chr4:57750017-57789643	39627	Duplication	*LOC255130*	*LOC255130*	1	0	0,698	Inf	na
4q26	chr4:114584724-114604318	19595	Deletion	*CAMK2D*	*CAMK2D*	1	0	0,698	Inf	na
4q27	chr4:120951145-121031754	80610	Deletion	*intergenic*	*intergenic*	1	0	0,698	Inf	na
4q28.3	chr4:135282185-135472380	190196	Deletion	*PABPC4L*	*PABPC4L*	1	0	0,698	Inf	na
4q28.3	chr4:136477938-136535637	57700	Deletion	*intergenic*	*intergenic*	1	0	0,698	Inf	na
4q34.3	chr4:180924994-180984457	59464	Duplication	*intergenic*	*intergenic*	1	0	0,698	Inf	na
5p15.2	chr5:9546723-10037618	490896	Duplication	*LOC285692,SEMA5A, SNORD123,TAS2R1*	*LOC285692,SEMA5A, SNORD123,TAS2R1*	1	0	0,698	Inf	na
5p12	chr5:44401302-44417455	16154	Deletion	*FGF10*	*intronic*	1	0	0,698	Inf	na
5q22.1	chr5:111015566-111030720	15155	Deletion	*LOC100505678*	*intronic*	1	0	0,698	Inf	na
5q22.3	chr5:113188389-113199827	11439	Deletion	*intergenic*	*intergenic*	1	0	0,698	Inf	na
6p25.1	chr6:5500587-5519318	18732	Deletion	*FARS2*	*intronic*	1	0	0,698	Inf	na
6q11.1	chr6:62247872-62263460	15589	Deletion	*intergenic*	*intergenic*	1	0	0,698	Inf	na
6q12	chr6:69294028-69298741	4714	Duplication	*intergenic*	*intergenic*	1	0	0,698	Inf	na
6q16.1	chr6:95424156-95654462	230307	Duplication	*intergenic*	*intergenic*	1	0	0,698	Inf	na
6q24.3	chr6:147657403-147677913	20511	Deletion	*STXBP5*	*STXBP5*	1	0	0,698	Inf	na
6q26	chr6:162077850-162413672	335823	Duplication	*PARK2*	*PARK2*	1	0	0,698	Inf	na
6q27	chr6:168092781-168153571	60791	Duplication	*HGC6.3,MLLT4*	*HGC6.3,MLLT4*	1	0	0,698	Inf	na
6q27	chr6:168240295-168334983	94689	Duplication	*intergenic*	*intergenic*	1	0	0,698	Inf	na
7p21.3	chr7:9094595-9184546	89952	Deletion	*intergenic*	*intergenic*	1	0	0,698	Inf	na
7p21.3	chr7:12759886-12788046	28161	Deletion	*intergenic*	*intergenic*	1	0	0,698	Inf	na
7p15.1	chr7:29175886-29503232	327347	Duplication	*CHN2*	*CHN2*	1	0	0,698	Inf	na
7p15.1	chr7:29657963-29738663	80701	Duplication	*DPY19L2P3,*	*DPY19L2P3,*	1	0	0,698	Inf	na

				LOC100271874, LOC646762,MIR550A3	*LOC100271874, LOC646762,MIR550A3*					
7p11.1	chr7:57495829-57524352	28524	Duplication	*ZNF716*	*ZNF716*	1	0	0,698	Inf	na
7q11.21	chr7:61726313-62326882	600570	Duplication	*intergenic*	*intergenic*	1	0	0,698	Inf	na
7q11.23	chr7:75955687-76046149	90463	Deletion	*DTX2,LOC100133091, UPK3B*	*DTX2,LOC100133091, UPK3B*	1	0	0,698	Inf	na
7q11.23	chr7:76126917-76395148	268232	Deletion	*intergenic*	*intergenic*	1	0	0,698	Inf	na
7q21.11	chr7:83368001-83386968	18968	Deletion	*intergenic*	*intergenic*	1	0	0,698	Inf	na
7q31.33	chr7:125937399-126382296	444898	Deletion	*GRM8*	*GRM8*	1	0	0,698	Inf	na
7q34	chr7:139716847-139754565	37719	Duplication	*RAB19,SLC37A3*	*RAB19,SLC37A3*	1	0	0,698	Inf	na
8p23.3	chr8:390600-401865	11266	Deletion	*FBXO25*	*FBXO25*	1	0	0,698	Inf	na
8p23.2	chr8:3104658-3115506	10849	Deletion	*CSMD1*	*intronic*	1	0	0,698	Inf	na
8p22	chr8:15447307-15453141	5835	Deletion	*TUSC3*	*intronic*	1	0	0,698	Inf	na
8p22	chr8:16010913-16070335	59423	Duplication	*MSR1*	*MSR1*	1	0	0,698	Inf	na
8p22	chr8:16460780-16569399	108620	Deletion	*intergenic*	*intergenic*	1	0	0,698	Inf	na
8p21.2	chr8:25588245-25613879	25635	Deletion	*intergenic*	*intergenic*	1	0	0,698	Inf	na
8q11.21	chr8:48397832-48770320	372489	Deletion	*KIAA0146*	*KIAA0146*	1	0	0,698	Inf	na
8q21.3	chr8:87314369-87346691	32323	Duplication	*intergenic*	*intergenic*	1	0	0,698	Inf	na
8q22.1	chr8:96549164-96574761	25598	Deletion	*intergenic*	*intergenic*	1	0	0,698	Inf	na
8q24.23	chr8:137799462-137917498	118037	Deletion	*intergenic*	*intergenic*	1	0	0,698	Inf	na
9p24.3	chr9:386380-468153	81774	Duplication	*DOCK8*	*DOCK8*	1	0	0,698	Inf	na
9p24.3	chr9:547617-702156	154540	Duplication	*KANK1*	*KANK1*	1	0	0,698	Inf	na
9p24.2	chr9:4133590-4216155	82566	Duplication	*GLIS3*	*GLIS3*	1	0	0,698	Inf	na
9p24.2	chr9:4313880-4536594	222715	Duplication	*SLC1A1*	*SLC1A1*	1	0	0,698	Inf	na
9p24.1	chr9:7710636-7726266	15631	Deletion	*intergenic*	*intergenic*	1	0	0,698	Inf	na
9p23	chr9:12275170-12387876	112707	Deletion	*intergenic*	*intergenic*	1	0	0,698	Inf	na
9p21.1	chr9:29564928-29578006	13079	Duplication	*intergenic*	*intergenic*	1	0	0,698	Inf	na
9p12	chr9:40772561-40795149	22589	Duplication	*ZNF658*	*ZNF658*	1	0	0,698	Inf	na

9q34.2	chr9:134939063-134947273	8211	Duplication	*intergenic*	*intergenic*	1	0	0,698	Inf	na
9q34.3	chr9:139662199-139714736	52538	Deletion	*EHMT1*	*intronic*	1	0	0,698	Inf	na
10p12.31	chr10:20836645-20896007	59363	Deletion	*MIR4675*	*MIR4675*	1	0	0,698	Inf	na
10q11.21	chr10:42130789-42147957	17169	Deletion	*LOC441666*	*LOC441666*	1	0	0,698	Inf	na
10q11.22	chr10:47066810-47122505	55696	Deletion	*intergenic*	*intergenic*	1	0	0,698	Inf	na
10q11.23	chr10:51743485-51778836	35352	Duplication	*SGMS1*	*SGMS1*	1	0	0,698	Inf	na
10q21.1	chr10:59142586-59157369	14784	Deletion	*intergenic*	*intergenic*	1	0	0,698	Inf	na
10q23.31	chr10:90935788-90967674	31887	Deletion	*CH25H,LIPA*	*CH25H,LIPA*	1	0	0,698	Inf	na
10q25.1	chr10:111192827-111223756	30930	Deletion	*intergenic*	*intergenic*	1	0	0,698	Inf	na
10q26.3	chr10:135108137-135215135	106999	Duplication	*CYP2E1,LOC619207*	*CYP2E1,LOC619207*	1	0	0,698	Inf	na
11p14.2	chr11:26975202-27192291	217090	Deletion	*BBOX1,FIBIN*	*BBOX1,FIBIN*	1	0	0,698	Inf	na
11p12	chr11:42371142-42390602	19461	Deletion	*intergenic*	*intergenic*	1	0	0,698	Inf	na
11q14.3	chr11:88173062-88197639	24578	Deletion	*GRM5*	*intronic*	1	0	0,698	Inf	na
11q22.1	chr11:99050585-99071992	21408	Deletion	*CNTN5*	*intronic*	1	0	0,698	Inf	na
12p13.33	chr12:2115897-2127756	11860	Deletion	*CACNA1C*	*intronic*	1	0	0,698	Inf	na
12p13.31	chr12:7760305-7813713	53409	Duplication	*CLEC4C,DPPA3, NANOGNB*	*CLEC4C,DPPA3, NANOGNB*	1	0	0,698	Inf	na
12p11.21	chr12:31903733-31945102	41370	Duplication	*intergenic*	*intergenic*	1	0	0,698	Inf	na
12q13.11	chr12:46754919-46788367	33449	Deletion	*PFKM,SENP1*	*PFKM,SENP1*	1	0	0,698	Inf	na
12q21.32	chr12:86033572-86059855	26284	Deletion	*intergenic*	*intergenic*	1	0	0,698	Inf	na
12q23.1	chr12:98810523-98978308	167786	Duplication	*ANKS1B,UHRF1BP1L*	*ANKS1B,UHRF1BP1L*	1	0	0,698	Inf	na
12q24.32	chr12:127794827-127820542	25716	Duplication	*intergenic*	*intergenic*	1	0	0,698	Inf	na
13q12.11	chr13:18775547-18801159	25613	Deletion	*ANKRD26P3*	*ANKRD26P3*	1	0	0,698	Inf	na
14q21.3	chr14:45221032-45408005	186974	Deletion	*intergenic*	*intergenic*	1	0	0,698	Inf	na
14q23.2	chr14:62851917-62869550	17634	Duplication	*GPHB5*	*GPHB5*	1	0	0,698	Inf	na
14q32.2	chr14:100339346-100351176	11831	Duplication	*MIR2392*	*MIR2392*	1	0	0,698	Inf	na
15q11.2	chr15:19416328-19535289	118962	Deletion	*LOC348120*	*LOC348120*	1	0	0,698	Inf	na
15q11.2	chr15:20648362-20667673	19312	Deletion	*LOC283683*	*LOC283683*	1	0	0,698	Inf	na

15q13.2	chr15:28737821-28751864	14044	Deletion	*LOC100288637*	*LOC100288637*	1	0	0,698	Inf	na
15q13.3	chr15:29827425-30300468	473044	Duplication	*CHRNA7*	*CHRNA7*	1	0	0,698	Inf	na
15q26.1	chr15:88554759-88615668	60910	Duplication	*C15orf58,CIB1, NGRN,SEMA4B,*	*C15orf58,CIB1, NGRN,SEMA4B,*	1	0	0,698	Inf	na
15q26.1	chr15:88685795-89010437	324643	Duplication	*TTLL13CRTC3, GABARAPL3,IQGAP1, ZNF774*	*TTLL13CRTC3, GABARAPL3,IQGAP1, ZNF774*	1	0	0,698	Inf	na
15q26.3	chr15:99865379-99923316	57938	Duplication	*intergenic*	*intergenic*	1	0	0,698	Inf	na
15q26.3	chr15:100052984-100105994	53011	Duplication	*TARSL2*	*TARSL2*	1	0	0,698	Inf	na
15q26.3	chr15:100182356-100215359	33004	Duplication	*GPCRLTM7*	*GPCRLTM7*	1	0	0,698	Inf	na
16p13.2	chr16:9109471-9161662	52192	Duplication	*C16orf72*	*C16orf72*	1	0	0,698	Inf	na
16p13.13	chr16:11397652-11543807	146156	Deletion	*intergenic*	*intergenic*	1	0	0,698	Inf	na
16p11.2	chr16:28733106-28825145	92040	Duplication	*ATP2A1,ATXN2L, MIR4721,RABEP2, SH2B1,TUFM*	*ATP2A1,ATXN2L, MIR4721,RABEP2, SH2B1,TUFM*	1	0	0,698	Inf	na
16p11.2	chr16:28895770-28933479	37710	Duplication	*LAT,SPNS1*	*LAT,SPNS1*	1	0	0,698	Inf	na
16q23.1	chr16:76607720-76622989	15270	Deletion	*CLEC3A*	*CLEC3A*	1	0	0,698	Inf	na
17p13.3	chr17:954290-1006780	52491	Duplication	*ABR*	*ABR*	1	0	0,698	Inf	na
17p13.3	chr17:1136077-1201625	65549	Duplication	*TUSC5,YWHAE*	*TUSC5,YWHAE*	1	0	0,698	Inf	na
17p13.1	chr17:9908739-9977572	68834	Deletion	*GAS7*	*NOT_FOUND*	1	0	0,698	Inf	na
17q12	chr17:30725132-30791060	65929	Deletion	*SLFN12,SLFN13*	*SLFN12,SLFN13*	1	0	0,698	Inf	na
17q12	chr17:31889664-31992082	102419	Duplication	*GGNBP2,MYO19, PIGW,ZNHIT3*	*GGNBP2,MYO19, PIGW,ZNHIT3*	1	0	0,698	Inf	na
17q12	chr17:32069090-32317483	248394	Duplication	*intergenic*	*intergenic*	1	0	0,698	Inf	na
17q12	chr17:32488438-33302932	814495	Duplication	*ACACA,C17orf78, DDX52,DUSP14, HNF1B,LOC284100, SYNRG,TADA2A*	*ACACA,C17orf78, DDX52,DUSP14, HNF1B,LOC284100, SYNRG,TADA2A*	1	0	0,698	Inf	na

17q21.31	chr17:38486384-38585342	98959	Deletion	*BRCA1,NBR1,NBR2*	*BRCA1,NBR1,NBR2*	1	0	0,698	Inf	na
17q25.1	chr17:69347869-69516090	168222	Duplication	*intergenic*	*intergenic*	1	0	0,698	Inf	na
17q25.1	chr17:69608379-69666897	58519	Duplication	*intergenic*	*intergenic*	1	0	0,698	Inf	na
17q25.1	chr17:69757224-69885849	128626	Duplication	*BTBD17,DNAI2, GPR142,KIF19,TTYH2*	*BTBD17,DNAI2, GPR142,KIF19,TTYH2*	1	0	0,698	Inf	na
17q25.1	chr17:69983674-70130820	147147	Duplication	*C17orf77,CD300A, CD300C,CD300E, CD300LB,CD300LD*	*C17orf77,CD300A, CD300C,CD300E, CD300LB,CD300LD*	1	0	0,698	Inf	na
18q22.1	chr18:62718985-62747566	28582	Duplication	*intergenic*	*intergenic*	1	0	0,698	Inf	na
19p12	chr19:20493452-20523385	29934	Deletion	*ZNF737*	*ZNF737*	1	0	0,698	Inf	na
19p12	chr19:20664930-20717774	52845	Duplication	*intergenic*	*intergenic*	1	0	0,698	Inf	na
19q13.31	chr19:48064226-48157011	92786	Deletion	*PSG1,PSG6,PSG7*	*PSG1,PSG6,PSG7*	1	0	0,698	Inf	na
19q13.41	chr19:58624107-58687562	63456	Duplication	*LOC147804, ZNF761,ZNF813*	*LOC147804, ZNF761,ZNF813*	1	0	0,698	Inf	na
20p12.1	chr20:14754577-14765018	10442	Deletion	*MACROD2*	*intronic*	1	0	0,698	Inf	na
20p11.21	chr20:23630133-23670319	40187	Duplication	*intergenic*	*intergenic*	1	0	0,698	Inf	na
20q11.21	chr20:29306843-29745884	439042	Duplication	*BCL2L1,COX4I2,DEFB115, DEFB116,DEFB118, DEFB119,DEFB121, DEFB122,DEFB123, DEFB124,HM13,ID1, MIR3193,NCRNA00028, PSIMCT-1,REM1*	*BCL2L1,COX4I2,DEFB115, DEFB116,DEFB118, DEFB119,DEFB121, DEFB122,DEFB123, DEFB124,HM13,ID1, MIR3193,NCRNA00028, PSIMCT-1,REM1*	1	0	0,698	Inf	na
21q21.1	chr21:19634654-19682955	48302	Deletion	*intergenic*	*intergenic*	1	0	0,698	Inf	na
21q22.12	chr21:36508747-36530895	22149	Duplication	*DOPEY2*	*DOPEY2*	1	0	0,698	Inf	na
22q11.22	chr22:21039509-21061758	22250	Deletion	*intergenic*	*intergenic*	1	0	0,698	Inf	na
22q11.23	chr22:24077300-24173884	96585	Duplication	*LRP5L*	*LRP5L*	1	0	0,698	Inf	na
15q14	chr15:32505886-32595143	89258	Deletion	*GOLGA8A*	*GOLGA8A*	8	4	0,728	0,85	na

8p23.2	chr8:5588599-5591903	3305	Deletion	*intergenic*	*intergenic*	6	3	0,732	0,85	na
2q37.3	chr2:242566407-242669396	102990	Deletion	*intergenic*	*intergenic*	4	2	0,745	0,86	na
17q21.31	chr17:41525626-41725112	199487	Duplication	*KIAA1267,LOC644246*	*KIAA1267,LOC644246*	4	2	0,745	0,86	na
1p36.33	chr1:1610720-1670079	59360	Duplication	*CDK11A,CDK11B, MMP23A,SLC35E2, SLC35E2B*	*CDK11A,CDK11B, MMP23A,SLC35E2, SLC35E2B*	2	1	0,784	0,86	na
4q32.2	chr4:162173179-162211190	38012	Duplication	*intergenic*	*intergenic*	2	1	0,784	0,86	na
11q22.3	chr11:107158291-107175438	17148	Duplication	*SLC35F2*	*SLC35F2*	2	1	0,784	0,86	na
12q24.33	chr12:130296270-130654881	358612	Deletion	*intergenic*	*intergenic*	2	1	0,784	0,86	na
16p13.3	chr16:2639305-2665257	25953	Duplication	*LOC100507321*	*LOC100507321*	2	1	0,784	0,86	na
17q12	chr17:31462326-31486394	24069	Duplication	*intergenic*	*intergenic*	2	1	0,784	0,86	na
2p11.2	chr2:88517095-89885025	1367931	Deletion	*ANKRD36BP2,C2orf51, EIF2AK3,FOXI3, MIR4436A,RPIA*	*ANKRD36BP2,C2orf51, EIF2AK3,FOXI3, MIR4436A,RPIA*	9	5	0,789	0,75	na
18q22.1	chr18:63441694-63819775	378082	Duplication	*LOC643542*	*LOC643542*	3	2	0,839	0,64	na
15q13.3	chr15:30297184-30302973	5790	Deletion	*intergenic*	*intergenic*	4	3	0,879	0,56	na
2p11.2	chr2:87297688-87730750	433063	Duplication	*MIR4435-1,MIR4435-2, NCRNA00152*	*MIR4435-1,MIR4435-2, NCRNA00152*	1	1	0,911	0,43	na
3p25.3	chr3:8795732-8832963	37232	Duplication	*intergenic*	*intergenic*	1	1	0,911	0,43	na
5p13.3	chr5:32144879-32192845	47967	Duplication	*GOLPH3,PDZD2*	*GOLPH3,PDZD2*	1	1	0,911	0,43	na
5q35.3	chr5:180109103-180122934	13832	Duplication	*intergenic*	*intergenic*	1	1	0,911	0,43	na
7q11.21	chr7:64316996-64578888	261893	Deletion	*INTS4L1,ZNF92*	*INTS4L1,ZNF92*	1	1	0,911	0,43	na
7q36.2	chr7:153158237-153351823	193587	Duplication	*DPP6*	*DPP6*	1	1	0,911	0,43	na
8p23.2	chr8:5237494-5276363	38870	Deletion	*intergenic*	*intergenic*	1	1	0,911	0,43	na
11q22.1	chr11:99428851-99459737	30887	Deletion	*CNTN5*	*CNTN5*	1	1	0,911	0,43	na
12q21.31	chr12:81696067-81727873	31807	Deletion	*TMTC2*	*intronic*	1	1	0,911	0,43	na
13q32.1	chr13:94797740-94825409	27670	Duplication	*intergenic*	*intergenic*	1	1	0,911	0,43	na
15q11.2	chr15:18811257-19170114	358858	Deletion	*GOLGA6L6,GOLGA8C,*	*GOLGA6L6,GOLGA8C,*	1	1	0,911	0,43	na

				HERC2P3,NBEAP1	*HERC2P3,NBEAP1*					
15q11.2	chr15:21937716-21984609	46894	Deletion	*PWRN2*	*PWRN2*	1	1	0,911	0,43	na
15q26.3	chr15:98730436-98770528	40093	Deletion	*LASS3*	*LASS3*	1	1	0,911	0,43	na
19p13.3	chr19:4826708-4861889	35182	Duplication	*ARRDC5,UHRF1*	*ARRDC5,UHRF1*	1	1	0,911	0,43	na
22q11.22	chr22:20644463-20903637	259175	Duplication	*TOP3B*	*TOP3B*	1	1	0,911	0,43	na
19q13.42	chr19:60018551-60069023	50473	Deletion	*KIR2DS4,KIR3DL1, KIR3DL2*	*KIR2DS4,KIR3DL1, KIR3DL2*	2	2	0,919	0,42	na
8q21.3	chr8:92192384-92252834	60451	Deletion	*LRRC69*	*LRRC69*	4	4	0,947	0,41	na
15q11.2	chr15:18788040-20077482	1289443	Duplication	*CXADRP2,GOLGA6L6, GOLGA8C,HERC2P3, LOC348120,LOC646214, LOC727924,NBEAP1, NF1P1,OR4M2,OR4N3P, OR4N4,POTEB,REREP3*	*CXADRP2,GOLGA6L6, GOLGA8C,HERC2P3, LOC348120,LOC646214, LOC727924,NBEAP1, NF1P1,OR4M2,OR4N3P, OR4N4,POTEB,REREP3*	12	9	0,949	0,51	na
5q21.1	chr5:101056643-101125626	68984	Duplication	*intergenic*	*intergenic*	2	3	0,972	0,27	na
2p22.3	chr2:35676878-35937068	260191	Deletion	*intergenic*	*intergenic*	1	2	0,974	0,21	na
18p11.32	chr18:1898848-1962346	63499	Deletion	*intergenic*	*intergenic*	1	2	0,974	0,21	na
22q11.23	chr22:24056992-24234257	177266	Deletion	*CRYBB2P1,LRP5L*	*CRYBB2P1,LRP5L*	1	2	0,974	0,21	na
1p21.1	chr1:105355887-105446771	90885	Deletion	*MIR548H3*	*intronic*	0	1	1,000	0,00	na
1q25.1	chr1:171328672-171372037	43366	Deletion	*intergenic*	*intergenic*	0	1	1,000	0,00	na
1q31.2	chr1:190131723-190142375	10653	Deletion	*intergenic*	*intergenic*	0	1	1,000	0,00	na
1q31.2	chr1:190619399-190639297	19899	Duplication	*intergenic*	*intergenic*	0	1	1,000	0,00	na
1q31.3	chr1:194918368-194951248	32881	Deletion	*CFH*	*CFH*	0	1	1,000	0,00	na
1q32.1	chr1:201975657-202015893	40237	Duplication	*ATP2B4,LAX1*	*ATP2B4,LAX1*	0	1	1,000	0,00	na
2p12	chr2:76936360-76972854	36495	Deletion	*LRRTM4*	*intronic*	0	1	1,000	0,00	na
2p11.2	chr2:88517095-88520452	3358	Deletion	*intergenic*	*intergenic*	0	1	1,000	0,00	na
2q23.3	chr2:154204396-154269871	65476	Deletion	*intergenic*	*intergenic*	0	1	1,000	0,00	na
3p22.2	chr3:37473214-37490345	17132	Duplication	*ITGA9*	*ITGA9*	0	1	1,000	0,00	na

3q29	chr3:193934412-193998204	63793	Duplication	*MB21D2*	*MB21D2*	0	1	1,000	0,00	na
4p16.3	chr4:2423474-2435102	11629	Duplication	*LOC402160*	*LOC402160*	0	1	1,000	0,00	na
4p12	chr4:47865426-47884898	19473	Duplication	*TEC*	*TEC*	0	1	1,000	0,00	na
4q32.1	chr4:160321804-160588615	266812	Duplication	*RAPGEF2*	*RAPGEF2*	0	1	1,000	0,00	na
4q32.3	chr4:164530235-164566231	35997	Deletion	*intergenic*	*intergenic*	0	1	1,000	0,00	na
5q35.3	chr5:178661436-178705647	44212	Duplication	*ADAMTS2*	*ADAMTS2*	0	1	1,000	0,00	na
6q16.1	chr6:95460371-95613585	153215	Deletion	*intergenic*	*intergenic*	0	1	1,000	0,00	na
6q23.3	chr6:137943136-137992740	49605	Duplication	*intergenic*	*intergenic*	0	1	1,000	0,00	na
7p21.3	chr7:12050906-12064226	13321	Deletion	*intergenic*	*intergenic*	0	1	1,000	0,00	na
7q11.21	chr7:64862630-64968436	105807	Duplication	*CCT6P1,LOC441242*	*CCT6P1,LOC441242*	0	1	1,000	0,00	na
7q21.3	chr7:97034347-97036072	1726	Duplication	*intergenic*	*intergenic*	0	1	1,000	0,00	na
7q31.1	chr7:110256224-110288215	31992	Deletion	*IMMP2L*	*intronic*	0	1	1,000	0,00	na
8p23.1	chr8:8149069-8182850	33782	Duplication	*intergenic*	*intergenic*	0	1	1,000	0,00	na
8p22	chr8:13645043-13663608	18566	Deletion	*intergenic*	*intergenic*	0	1	1,000	0,00	na
8q12.1	chr8:60909551-61253195	343645	Duplication	*intergenic*	*intergenic*	0	1	1,000	0,00	na
8q24.21	chr8:128573181-128595167	21987	Deletion	*intergenic*	*intergenic*	0	1	1,000	0,00	na
8q24.3	chr8:146116506-146189313	72808	Deletion	*ZNF16,ZNF252*	*ZNF16,ZNF252*	0	1	1,000	0,00	na
10q11.21	chr10:44538847-44641858	103012	Duplication	*LOC220980*	*LOC220980*	0	1	1,000	0,00	na
10q21.3	chr10:67866550-68091312	224763	Deletion	*CTNNA3*	*CTNNA3*	0	1	1,000	0,00	na
10q22.3	chr10:81633431-81660920	27490	Duplication	*LOC100288974*	*LOC100288974*	0	1	1,000	0,00	na
10q22.3	chr10:81702420-81757368	54949	Duplication	*intergenic*	*intergenic*	0	1	1,000	0,00	na
10q22.3	chr10:81802016-81849467	47452	Duplication	*C10orf57,LOC219347*	*C10orf57,LOC219347*	0	1	1,000	0,00	na
10q22.3	chr10:81884474-81902159	17686	Duplication	*PLAC9*	*PLAC9*	0	1	1,000	0,00	na
10q24.2	chr10:100467375-100542446	75072	Deletion	*HPSE2*	*HPSE2*	0	1	1,000	0,00	na
10q26.12	chr10:122919651-122939496	19846	Deletion	*intergenic*	*intergenic*	0	1	1,000	0,00	na
11p14.1	chr11:30716911-30737507	20597	Duplication	*intergenic*	*intergenic*	0	1	1,000	0,00	na
11p13	chr11:34607340-34727070	119731	Duplication	*EHF*	*EHF*	0	1	1,000	0,00	na
11p11.2	chr11:48546574-48621207	74634	Deletion	*intergenic*	*intergenic*	0	1	1,000	0,00	na

11q22.2	chr11:102278008-102326614	48607	Deletion	*MMP13*	*MMP13*	0	1	1,000	0,00	na
13q32.1	chr13:94740493-94764852	24360	Duplication	*ABCC4*	*ABCC4*	0	1	1,000	0,00	na
14q23.1	chr14:59651616-59703483	51868	Duplication	*C14orf135,DHRS7*	*C14orf135,DHRS7*	0	1	1,000	0,00	na
14q32.33	chr14:105714823-105780975	66153	Deletion	*intergenic*	*intergenic*	0	1	1,000	0,00	na
15q13.3	chr15:30297184-30302973	5790	Duplication	*intergenic*	*intergenic*	0	1	1,000	0,00	na
15q23	chr15:66422629-66472024	49396	Duplication	*ITGA11*	*ITGA11*	0	1	1,000	0,00	na
15q25.3	chr15:85634145-85671028	36884	Deletion	*intergenic*	*intergenic*	0	1	1,000	0,00	na
16p13.11	chr16:15631614-15790710	159097	Duplication	*KIAA0430,MIR484, MYH11,NDE1*	*KIAA0430,MIR484, MYH11,NDE1*	0	1	1,000	0,00	na
16p13.11	chr16:15900900-15944762	43863	Duplication	*intergenic*	*intergenic*	0	1	1,000	0,00	na
16p13.11	chr16:16031509-16065535	34027	Duplication	*ABCC1*	*ABCC1*	0	1	1,000	0,00	na
16p13.11	chr16:16088643-16094970	6328	Duplication	*ABCC1*	*ABCC1*	0	1	1,000	0,00	na
16p13.11	chr16:16178405-16197033	18629	Duplication	*ABCC6*	*ABCC6*	0	1	1,000	0,00	na
16p12.3	chr16:16817634-16958375	140742	Duplication	*intergenic*	*intergenic*	0	1	1,000	0,00	na
16p12.3	chr16:17075737-17176986	101250	Duplication	*XYLT1*	*XYLT1*	0	1	1,000	0,00	na
16p12.3	chr16:17541215-17773612	232398	Duplication	*intergenic*	*intergenic*	0	1	1,000	0,00	na
16p12.3	chr16:17942205-18063852	121648	Duplication	*intergenic*	*intergenic*	0	1	1,000	0,00	na
16p11.2	chr16:34332760-34393084	60325	Duplication	*intergenic*	*intergenic*	0	1	1,000	0,00	na
18q22.1	chr18:62256840-62485883	229044	Deletion	*CDH19*	*CDH19*	0	1	1,000	0,00	na
19p13.3	chr19:3755701-3779433	23733	Duplication	*ZFR2*	*ZFR2*	0	1	1,000	0,00	na
19q12	chr19:33063516-33142870	79355	Deletion	*intergenic*	*intergenic*	0	1	1,000	0,00	na
22q11.1	chr22:15599625-15826991	227367	Duplication	*GAB4,HSFY1P1,XKR3*	*GAB4,HSFY1P1,XKR3*	0	1	1,000	0,00	na

Abbreviations: CI = confidence interval; HBOC = hereditary breast and/or ovarian cancer; ind = individual; na = not available; no = number; OR = odds ratio.

[a]According to the NCBI Genome Build 36 (hg 18).
