# Supplementary material for: Copy Number Variation Analysis in Familial BRCA1/2-Negative Finnish Breast and Ovarian Cancer
Source: PLoS One. 2013 Aug 13;8(8):e71802. doi: 10.1371/journal.pone.0071802 (PMC3742470; doi:10.1371/journal.pone.0071802)
Supplement: Table S2 — Enriched GO term categories and pathways ( P -value less than 0.05) involving EPHA3 , ERBB4 and BRCA1. (PDF) [file pone.0071802.s003.pdf]

Table S2. Enriched GO term categories and pathways (*P*-value less than 0.05) involving *EPHA3*, *ERBB4* and *BRCA1*.

***EPHA3*-and *ERBB4*-related molecular functions by GO analysis**	**GO term/pathway id**
Receptor activity	GO:0004872
Transmembrane receptor activity	GO:0004888
Molecular transducer activity	GO:0060089
Signal transducer activity	GO:0004871
***EPHA3*-related biological processes by GO analysis**	
multi-organism process	GO:0051704
***EPHA3*-related pathways by KEGG pathway analysis**	
Axon guidance	KEGG ID:4360
***ERBB4*-related pathways by KEGG pathway analysis**	
Calcium signaling pathway	KEGG ID:4020
***BRCA1*-related pathways by pathway commons analysis**	
Homologous recombination repair of replication-independent double-strand breaks	DB_ID:433
ATM mediated response to DNA double-strand break	DB_ID:435
Homologous Recombination Repair	DB_ID:432
Double-Strand Break Repair	DB_ID:767

Enrichment analysis was performed using the Web-based Gene Set Analysis Toolkit V2 (WebGestalt2) [1].

References

1. Zhang B, Kirov S, Snoddy J. (2005) WebGestalt: An integrated system for exploring gene sets in various biological contexts. Nucleic Acids Res 33: W741-8.
