# Supplementary material for: Copy Number Variation Analysis in Familial BRCA1/2-Negative Finnish Breast and Ovarian Cancer
Source: PLoS One. 2013 Aug 13;8(8):e71802. doi: 10.1371/journal.pone.0071802 (PMC3742470; doi:10.1371/journal.pone.0071802)
Supplement: File S1 — Clinical characteristics of three additional individuals. (PDF) [file pone.0071802.s004.pdf]

**File S1. Clinical characteristics of three additional individuals.**

Three additional individuals had the following characteristics: Patient 252 (family id) had ductal grade 3, estrogen receptor (ER) and progesterone receptor (PR) negative and human epidermal growth factor 2 (HER2) positive breast cancer diagnosed at age 50 years. Two of her mother's sisters had breast cancer diagnosed at ages of 36 and 39 years respectively (according to the patient information). The second individual (family id 201) was healthy but her mother had breast cancer diagnosed at age 41 years and mother's sister had breast cancer diagnosed at age 65 years (patient information). Third individual (family id 217) was healthy but her mother had been diagnosed ovarian cancer before age 59 years and mother's sister had breast cancer diagnosed at age 61 years. Additionally, two of her maternal female cousins had breast cancers of which one was bilateral and diagnosed before age 40 years (patient information).
