# Supplementary material for: Copy Number Variation Analysis in Familial BRCA1/2-Negative Finnish Breast and Ovarian Cancer
Source: PLoS One. 2013 Aug 13;8(8):e71802. doi: 10.1371/journal.pone.0071802 (PMC3742470; doi:10.1371/journal.pone.0071802)
Supplement: File S2 — Clinical characteristics of 20 additional HBOC individuals utilised for CNV validation analysis. (PDF) [file pone.0071802.s005.pdf]

File S2. Clinical characteristics of 20 additional HBOC individuals utilised for CNV validation analysis.

Family	Cancer (age at diagnosis)	Breast / Ovarian cancer histology/grade	Receptor status	Breast / Ovarian cancer cases in the family (age at diagnosis if known)
TuFamBC1	Breast (38)	ductal, gr 3	ER+, PR+, HER2+	Maternal side of the family: Bilateral Breast (61), 2 x Breast (36 and 59)
TuFamBC2	Breast (41)	ductal, gr 2	ER+, PR+, HER2+	Maternal side of the family: 3x Breast (60, 60, 66)
TuFamBC3	Breast (28)	ductal, gr 3	ER-, PR-, HER2-	Maternal side of the family: Breast (45)
TuFamBC4	Breast (32)	ductal, gr 3	ER-, PR+, HER2-	Maternal side of the family: 2 x Breast (early onset, 60)
TuFamBC5	Breast (32)	ductal, gr 1	ER+, PR+, HER2-	Maternal side of the family: Breast (70); Sister: Breast (44 and 49)
TuFamBC6	Bilateral Breast (40)	ductal, gr 1 and ductal, gr 3	ER+, PR+, HER2-	-
TuFamBC7[a]	Breast (36)	ductal, na	ER+, PR+, HER2-	Maternal side of the family: Breast (55)
TuFamBC8[a]	Breast (28)	ductal, gr 3	ER+, PR+, HER2+	Maternal side of the family: 2 x Breast; Paternal side of the family: Breast (78)
TuFamBC9	Bilateral Breast (44)	ductal, na	ER+, PR+, HER2-	Sister: Breast (58)
TuFamBC10	Breast (49)	ductal, gr 3	ER+, PR+, HER2+	Sister: Bilateral Breast (43)
TuFamBC11	Breast (53)	ductal, gr 1	ER+, PR+, HER2-	Maternal side of the family: Breast (53); Sister: Breast (42)
TuFamBC12[a, c]	Breast (66)	ductal, gr 2	ER+, PR+, HER2-	Sisters: Breast (61), 2x Ovarian (67, 78); Paternal side of the family: Breast
TuFamBC13[c]	Ovarian (61)	serous papillary, gr 3	-	Sister: Breast (50)
TuFamBC14	Breast (50)	ductal, gr 2	ER+, PR+, na	Maternal side of the family: Breast (35)
TuFamBC15[b]	Breast (46)	ductal, gr 3	ER+, PR+, HER2-	Sister: Breast (36)
TuFamBC16	Breast (74)	ductal, gr 2	ER+, PR+, HER2-	Maternal side of the family: Breast (80), Ovarian (75); Sisters: Bilateral Breast (57), Ovarian (78)
TuFamBC17	Bilateral Breast (35)	ductal, gr 1	ER+, PR+, na	Maternal side of the family: Breast, Paternal side of the family: Breast
TuFamBC18[c]	Bilateral Breast (50, 70), Ovarian (60)	ductal, gr 1	ER+, PR+, HER2-	Maternal side of the family: Breast
TuFamBC19	Breast (48)	lobular, gr 1	ER+, PR+, HER2-	Maternal side of the family: Breast (57)
TuFamBC20[c]	Breast (52)	ductal, gr 1	ER+, PR+, HER2-	Maternal side of the family: 2 x Breast (45, 50), Ovarian (45); Sisters: 2 x Breast (52 and 58),

Abbreviations: ER = estrogen receptor; gr = grade; HER2 = human epidermal growth factor receptor 2; na = not available; PR = progesterone receptor.
[a] Heterozygous duplication in the 19q13.41 region (copy number 3). [b] Homozygous duplication in the 19q13.41 region (copy number 4). [c] Heterozygous deletion in the 3p11.1 region (copy number 1).
