# Supplementary material for: Copy Number Variation Analysis in Familial BRCA1/2-Negative Finnish Breast and Ovarian Cancer
Source: PLoS One. 2013 Aug 13;8(8):e71802. doi: 10.1371/journal.pone.0071802 (PMC3742470; doi:10.1371/journal.pone.0071802)
Supplement: File S3 — Copy number variation validation protocol by quantitative RT-PCR. (PDF) [file pone.0071802.s006.pdf]

File S3. Copy number variation validation protocol by quantitative RT-PCR.

Amplification reactions (10 µl) were performed in quadruplicate with 10 ng of template DNA, 2X TaqMan® Genotyping Master Mix (Applied Biosystems, Foster City, CA, USA), 900 nM of each primer and 250 nM of each fluorogenic probe. Thermal cycling conditions were: 2 mins at 50° C, 10 mins at 95° C, followed by 40 cycles of 15 secs at 95° C and 60 secs at 60° C. Real-time data was collected by SDS 2.2.2 software (Applied Biosystems) and analyzed by the Copy Caller v.1.0 software (Applied Biosystems). Comparative C_T method (Applied Biosystems user bulletin Part number 4400042) was used to calculate copy numbers. Each replicate was normalized to RNAseP to get ΔC_T (FAM dye C_T – VIC dye C_T). Average ΔC_T for each sample was calculated from the four replicates. All of the samples were normalized to a calibrator sample to determine $\Delta\Delta C_T$. Copy number was obtained from the formula $2 \times 2^{-\Delta\Delta C_T}$. Each experiment was repeated three times.
